# Pseudomonas infections among hospitalized adults in Latin America: a systematic review and meta-analysis

**DOI:** 10.1186/s12879-020-04973-0

**Published:** 2020-03-27

**Authors:** Alfredo Ponce de Leon, Sanjay Merchant, Gowri Raman, Esther Avendano, Jeffrey Chan, Griselda Tepichin Hernandez, Eric Sarpong

**Affiliations:** 1 Department of Infectious Diseases, Laboratory of Clinical Microbiology, Instituto Nacional de Ciencias Médicas y Nutrición Salvador Zubirán, Mexico City, Mexico; 2 Merck & Co., Inc, Kenilworth, NJ USA; 3 Institute for Clinical Research and Health Policy Studies, Center for Clinical Evidence Synthesis, Tufts Medical Center, 800 Washington St, Boston, MA 02111 USA; 4 Tufts University School of Medicine, Boston, MA USA

**Keywords:** *Pseudomonas aeruginosa*, Appropriate, Inappropriate, Latin America, Risk factors, Antibiotic therapy, Resistance

## Abstract

**Background:**

Treatment of resistant *Pseudomonas aeruginosa* infection continues to be a challenge in Latin American countries (LATAM). We synthesize the literature on the use of appropriate initial antibiotic therapy (AIAT) and inappropriate initial antibiotic therapy (IIAT) in *P. aeruginosa* infections, and the literature on risk factors for acquisition of resistant *P. aeruginosa* among hospitalized adult patients in LATAM.

**Methods:**

MEDLINE, EMBASE, Cochrane, and LILAC were searched between 2000 and August 2019. Abstracts and full-text articles were screened in duplicate. Random effects meta-analysis was conducted when studies were sufficiently similar.

**Results:**

The screening of 165 citations identified through literature search yielded 98 full-text articles that were retrieved and assessed for eligibility, and 19 articles conducted in Brazil (14 articles), Colombia (4 articles), and Cuba (1 article) met the inclusion criteria. Of 19 eligible articles, six articles (840 subjects) examined AIAT compared to IIAT in *P. aeruginosa* infections; 17 articles (3203 total subjects) examined risk factors for acquisition of resistant *P. aeruginosa*; and four articles evaluated both. Four of 19 articles were rated low risk of bias and the remaining were deemed unclear or high risk of bias. In meta-analysis, AIAT was associated with lower mortality for *P. aeruginosa* infections (unadjusted summary OR 0.48, 95% CI 0.28–0.81; I^2^ = 59%), compared to IIAT and the association with mortality persisted in subgroup meta-analysis by low risk of bias (3 articles; unadjusted summary OR 0.46, 95% CI 0.28–0.81; I^2^ = 0%). No meta-analysis was performed for studies evaluating risk factors for acquisition of resistant *P. aeruginosa* as they were not sufficiently similar. Significant risk factors for acquisition of resistant *P. aeruginosa* included: prior use of antibiotics (11 articles), stay in the intensive care unit (ICU) (3 articles), and comorbidity score (3 articles). Outcomes were graded to be of low strength of evidence owing to unclear or high risk of bias and imprecise estimates.

**Conclusion:**

Our study highlights the association of AIAT with lower mortality and prior use of antibiotics significantly predicts acquiring resistant *P. aeruginosa* infections. This review reinforces the need for rigorous and structured antimicrobial stewardship programs in the LATAM region*.*

## Background

Antimicrobial resistant Gram-negative infections continue to increase and pose a major public health problem in LATAM [1, 2]. Analysis of 245 *P. aeruginosa* isolates from a 2018 Program to Assess Ceftolozane/Tazobactam Susceptibility (PACTS) database on 6 countries (Argentina, Brazil, Chile, Costa Rica, Mexico, and Panama) highlight the relatively high rates of antimicrobial resistance in Latin America (Table 1) [3, 4]. Among Gram-negative pathogens, resistant *P. aeruginosa* is the most common cause of nosocomial and healthcare associated infections (HAIs) [2]. Resistant *P. aeruginosa* is associated with increased mortality and significant costs [3]. The treatment of resistant *P. aeruginosa*, an opportunistic pathogen with an ability to rapidly develop resistance to multiple classes of antibiotics, is especially challenging.

**Table 1:** Susceptibility of *P. aeruginosa* (245 isolates) Pathogens to Antimicrobials in the Latin America region^a^ from the 2018 PACTS^b^ Database

				% Susceptible					
	MIC (mg/mL)		Range	CLSI^**c**^			EUCAST^**c**^		
**Antimicrobial agent**	**50%**	**90%**		**%S**	**%I**	**%R**	**%S**	**%I**	**%R**
Ceftolozane-tazobactam	0.5	4	0.12 to > 32	90.2	0.8	9.0	90.2		9.8
Amikacin	4	> 32	0.5 to > 32	83.6	1.2	15.2	79.1	4.5	16.4
Ampicillin-sulbactam	> 64	> 64	8 to > 64						
Aztreonam	8	> 16	0.25 to > 16	64.1	13.5	22.4	77.6		22.4
Cefepime	2	32	0.25 to > 256	80.0	7.8	12.2	80.0		20.0
Ceftazidime	2	> 32	0.5 to > 32	77.1	6.1	16.7	77.1		22.9
Ceftriaxone	> 8	> 8	1 to > 8						
Ciprofloxacin	0.12	> 16	≤0.03 to > 16	68.9	4.5	26.6	68.9		31.1
Colistin	0.5	1	≤0.06 to 2	100.0		0.0	100.0		0.0
Doripenem	0.5	8	≤0.06 to > 8	78.4	7.3	14.3	72.2	6.1	21.6
Gentamicin	2	> 16	≤0.12 to > 16	77.0	2.0	20.9	77.0		23.0
Imipenem	1	> 8	≤0.12 to > 8	74.3	3.7	22.0	78.0		22.0
Levofloxacin	0.5	32	0.03 to > 32	64.3	5.7	29.9	64.3		35.7
Meropenem	0.5	16	≤0.015 to > 32	73.5	7.8	18.8	73.5	12.2	14.3
Piperacillin-tazobactam	4	128	0.25 to > 128	74.3	12.2	13.5	74.3		25.7
Tigecycline	8	> 8	1 to > 8						

^a^ Includes only Latin American countries (i.e., Argentina, Brazil, Chile, Costa Rica, Mexico, and Panama) with available data in the PACTS database

^b^*PACTS* Program to Assess Ceftolozane/Tazobactam Susceptibility

^c^ Criteria as published by CLSI [2019] and EUCAST [2019]

*CLSI* Clinical and Laboratory Standards Institute; *EUCAST* European Committee on Antimicrobial Susceptibility Testing; *MIC* minimum inhibitory concentration; *PACTS* Program to Assess Ceftolozane/Tazobactam Susceptibility

IIAT in pneumonia, sepsis, and other infections can adversely impact health outcomes, increase length of stay in the hospital, and can result in increased mortality [5]. Identifying factors predicting the risk for acquisition of resistant *P. aeruginosa* or identifying subgroups of patients who are at an increased risk for such acquisition can help facilitate surveillance and optimize therapeutic management. Additionally, the long-term effectiveness of antimicrobials is reliant on appropriate and controlled use, and effective antibiotic stewardship, as stipulated in current guidelines [6]. There is a considerable gap in knowledge regarding the role of IIAT use in the LATAM region as well as risk factors associated with occurrence of resistant *P. aeruginosa* in hospitalized patients in the LATAM region.

Although available data suggest that AIAT reduce mortality and improve outcomes, to our knowledge, no study has systematically synthesized their specific contribution in LATAM. A number of individual studies exist in the public domain demonstrating consequences of AIAT versus IIAT as well as those that examine risk factors associated with resistant *P. aeruginosa* infections in LATAM. However, there has not been a comprehensive and systematic evaluation of the contemporary literature on this topic among hospitalized adult patients in LATAM.

This systematic literature review focuses on LATAM countries and critically examines two objectives: 1) the role of AIAT, as compared with IIAT in hospitalized adult patients undergoing initial treatment for nosocomial or hospital-acquired or healthcare-associated *P. aeruginosa* infections; 2) examine available evidence on risk factors associated with acquisition of resistant *P. aeruginosa* infection among hospitalized adult patients.

## Methods

We followed standard systematic review methods of conduct and reporting as detailed in the Preferred Reporting Items for Systematic Reviews and Meta-Analyses (PRISMA) criteria [7]. A priori protocol was created and retained for internal reference.

### Search strategy

We performed a comprehensive systematic literature search in the MEDLINE®, Cochrane Central, and EMBASE® databases for citations indexed from January 2000 through August 2019 without any language restriction. Additional searches were conducted in the Literatura Latino Americana em Ciências da Saúde (LILAC) and Biblioteca Regional de Medicina (BIREME) databases for citations indexed from January 2000 to August 2019 to identify Spanish and other foreign language articles from the LATAM region. We searched for contemporary articles in this area beginning from 2000 because a previous study that evaluated global publications on this topic identified articles published from mid-to-late 2000 onwards [8]. To ensure completeness of our systematic literature search, we reviewed reference lists of eligible studies and eligible systematic reviews identified from the aforementioned sources. We sought input from an infectious disease clinical expert in the LATAM region with regard to any potentially eligible publications. When eligible, we also considered abstracts from conference proceedings. Separate searches were conducted for each of the objectives. Supplemental Table 1 lists the search strategy terms related to the pathogen (*P. aeruginosa*), site of infection (urinary tract, intra-abdominal, bloodstream, and pneumonia), initial therapy (inappropriate, appropriate, adequate, or effective), and antibacterial drug therapy. Supplemental Table 2 lists terms related to *P. aeruginosa*, mode of infection (nosocomial, hospital-acquired, healthcare-acquired, hospital-associated, healthcare-associated), and risk factor assessment (risk factors, predict, risk score, risk assessment, and multivariate analysis) and was limited to the LATAM region (Latin America and Caribbean countries). Articles published in Spanish and Portuguese languages, the most common languages from the LATAM region were translated by one of the authors (EEA).

### Study selection

We screened all citations in duplicate. During initial rounds of citation screening, we implemented a training session where all researchers screened the same set of articles and iteratively continued training until all researchers agreed with the nuances of citation screening and selection. Disagreements were resolved in group meetings in discussion with a senior reviewer.

#### Study eligibility criteria

We included articles evaluating adults ≥18 years of age hospitalized in a ward, intensive or critical care unit with confirmed acquisition of nosocomial or hospital-acquired or healthcare-associated *P. aeruginosa* infection. For studies with multiple publications, we included those with the longest follow-up, largest sample size, or both. Eligible data from observational (prospective and retrospective studies) and trials were included.

We excluded studies that were conducted among pediatric population. International studies that did not report stratified data by LATAM region were excluded. Non-human studies, narrative reviews, cross-sectional studies, case reports, editorials, letters and notes/comments were also excluded. In addition to the above common eligibility criteria, we specified the following additional criteria according to each of the objectives:

#### Study eligibility criteria for AIAT vs. IIAT

Studies of adult patients with susceptible, resistant, or MDR *P. aeruginosa* infections of the following sites: respiratory, intra-abdominal, bloodstream, and urinary tract were included. Studies that compared patients who received AIAT and IIAT and reported outcomes of interest were included. The outcomes of interest included: mortality (primary outcome), clinical success, microbiologic eradication, length of stay (hospital or ICU), and cost.

Studies with patients that may have been exposed to initial antimicrobial therapy before being hospitalized were excluded.

#### Study eligibility criteria for risk factors for acquiring resistant *P. aeruginosa*

Articles reporting risk factors that predicted acquisition of resistant *P. aeruginosa*, with or without extended-spectrum beta-lactamases (ESBLs) were included. Resistant *P. aeruginosa* infection was defined as cephalosporin-resistant and/or piperacillin/tazobactam-resistant and/or carbapenem-resistant, and/or multi-drug (MDR) or and/or extremely drug-resistant (XDR) *P. aeruginosa* infection. Studies reporting any of the following comparisons were included: MDR/XDR versus resistant *P. aeruginosa*, MDR/XDR/resistant versus susceptible *P. aeruginosa,* and MDR/XDR/resistant versus any control.

### Data collection

All articles identified as possibly meeting the eligibility criteria were then extracted independently by one experienced reviewer and the data were then validated by a second reviewer.

Pertinent data including study design, country where study was conducted, funding source, participant characteristics, identified pathogen, source of infection, site of infection, inclusion criteria, exclusion criteria, co-occurring conditions, co-morbidity scores, and unadjusted or adjusted analysis were extracted into customized forms in Excel®. Data extraction for AIAT versus IIAT included author’s definition of AIAT or IIAT (individual study definitions of AIAT and IIAT were accepted), percentage of patients receiving AIAT or IIAT. We tested the data extraction forms on several studies and revised as necessary before full data extraction. Any missing information was deemed as not reported information. Any disagreements were resolved by discussion amongst the team to achieve consensus.

### Assessment of study quality

We assessed the methodological quality of each study based on predefined criteria. We used the Agency for Healthcare Research and Quality (AHRQ) risk of bias tool, which asks about risk of selection bias, performance bias, detection bias, attrition bias, reporting bias, and other potential biases [9]. Risk of bias was assessed by two reviewers and disagreements were resolved in group meetings in discussion with a senior reviewer.

### Assessment of overall strength of evidence

The Grading of Recommendations Assessment, Development and Evaluation (GRADE; https://www.gradeworkinggroup.org/) was employed to assess the strength of overall evidence for outcomes that were assessed in meta-analysis and graded into one of the following: High, Moderate, Low or Very Low. The GRADE assessments were conducted in duplicate and disagreements were resolved in consensus.

### Data analysis

When eligible studies were clinically heterogeneous in terms of outcomes or comparators, data were presented descriptively using systematic review instead of meta-analysis. We conducted meta-analyses of eligible studies that assessed sufficiently similar populations and outcomes and presented results of random effects meta-analysis as this model can estimate the mean of a distribution of true effect size (ES) [10]. For unadjusted results evaluating AIAT versus IIAT, we estimated odds ratio (OR) and its 95% confidence interval (CI) from data reported in individual studies. If available, we combined adjusted OR across studies. We tested between-study statistical heterogeneity with the Q statistic (*p* < 0.10 was deemed statistically significant) and quantified its extent with I^2^ and 95% CI [11]. We conducted analyses in Stata version 14 (StataCorp, College Station, Texas) with the metan, metareg, and metabias functions. For risk factors for acquiring *resistant P. aeruginosa,* we evaluated results reported in multivariable analyses that adjusted for potential confounders.

## Results

### Baseline study and population characteristics

**Fig. 1:**
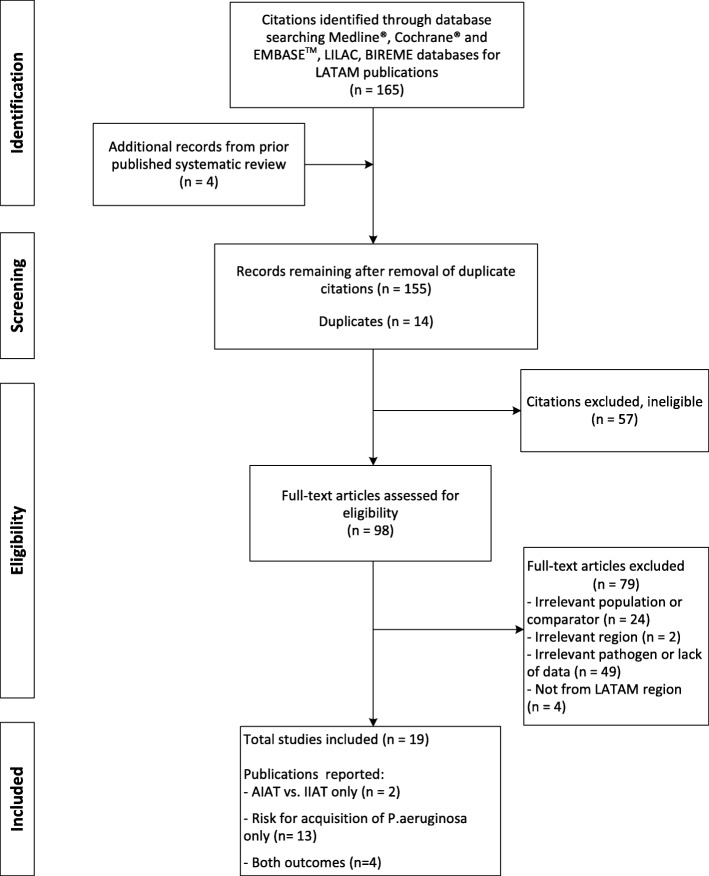
PRISMA Flow Diagram

The literature search identified a total of 165 citations; of which, 98 full-text articles were retrieved and assessed for eligibility. The systematic review included 19 articles (6 articles for AIAT versus IIAT [12–17]; 17 articles for acquisition of resistant or multidrug-resistant *P. aeruginosa* [12, 14–16, 18–30] and 4 articles reported both outcomes [12, 14–16]) that met final eligibility criteria (Fig. 1).

### AIAT vs. IIAT

Seven eligible studies (published in six articles) included 885 hospitalized adults with *P. aeruginosa* infections. Included studies were conducted between 2005 and 2012 in Brazil (5 studies [12, 14, 15, 17, 31]) and Colombia (1 study). One study was retrospective cohort and five studies were case-control designs (Table 2). The underlying site of infection was bloodstream infection (3 studies [12, 15, 31]), respiratory (2 studies [13, 17]) and information was not reported in one study [14]. The average age of patients among included studies ranged between 50 and 73 years.

**Table 2:** Summary baseline table of studies comparing AIAT vs. IIAT

Author Year	Country (Enrollment Period)	Study Design	Total Follow-up	Total N	Pathogen	Site of Infection	Mean Age (SD), yr	% Male	% IIAT	Timeliness of AIAT	Susceptibility reported
Araujo 2016 [12]	Brazil (2009–2012; 2014–2014)	CC	~ 55 days	236	MDR and Non-MDR PA	BSI: 100%	52.7 (22.9)	70.3	36	< 24 h	Yes
Dantas 2014 [15]	Brazil (2009–2011)	RC	~ 55.4 days	120	Resistant and susceptible PA	BSI: 100%	51.5 (3.2)	63.3	28.3	NR	NR
Gonzales 2014 [13]	Colombia (2005–2008)	RC; CC	30 days	164	PA	RS: 37.5%; Central venous catheter 28.6%	56 (33.5)	67.1	50	< 48 h	Yes
Pinheiro 2008 [17]	Brazil (2006–2007)	RC; CC	30 days	131	PA	RS: 65.6%; BSI: 18.3%; UTI: 11.5%	64.2 (18.4)	50.4	37.3	NR	Yes
Tuon 2012 [16]	Brazil (2006–2009)	CC	30 days	77	CRPA, CSPA	BSI: 100%	48.0	72.7	52	< 24 h	Yes
Rossi 2017 [14]	Brazil (2009–2012)	CC	NR	157	PA 100%	Unknown: 62.42; RS: 17.19; BSI: 13.37	52.0 (24.5)	66.9	31.2	NR	Yes

*AIAT* Appropriate initial antibiotic therapy; *BSI* Bacteremia/Bloodstream infection; *CC* Case-control; *CRPA* Carbapenem-resistant *Pseudomonas aeruginosa*; *CSPA* Carbapenem-susceptible *Pseudomonas aeruginosa*; *H* hour; *IIAT* Inappropriate initial antibiotic therapy; *MDR* Multidrug resistant; *N* Number; *NR* Not reported; *PA Pseudomonas aeruginosa*; *RC* Retrospective; *RS* Respiratory; *UTI* Urinary tract infection; *Yr* Year

Included studies heterogeneously defined appropriate use of antibiotic therapy. The common definition was use of initial antimicrobial agent within a specific number of hours (between 24 and < 48 h) after index blood culture and/or that initial antibiotic displayed susceptibility against pathogen of interest in subsequent in vitro examination (Table 2). All studies reported 30-day mortality data and no studies reported data on treatment response, cost, or length of stay outcomes. The risk of bias was, generally, high or unclear. For example, most of these studies failed to clearly report the methods employed (Supplemental Table 3).

### Mortality outcomes in AIAT vs. IIAT

#### Unadjusted data

**Fig. 2:**
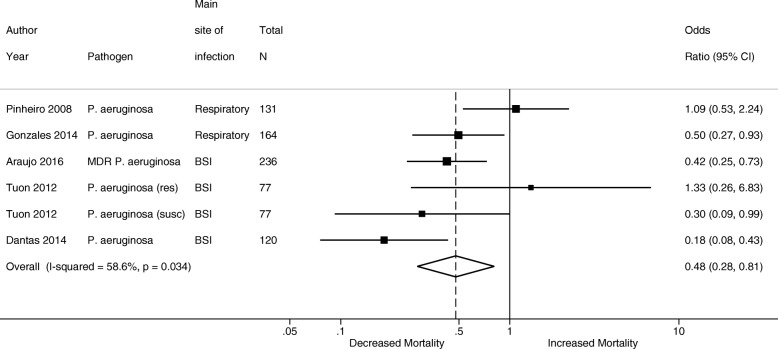
Meta-analysis of Mortality among AIAT compared with IIAT in *P. aeruginosa.* Figure 2 identifies a significantly decreased mortality with AIAT vs. IIAT for the subgroup of *P. aeruginosa.* AIAT = Appropriate initial antibiotic therapy; IIAT = Inappropriate initial antibiotic therapy; BSI = Bacteremia/Blood stream infection; MDR: Multidrug resistant; N: Number; *P. aeruginosa* = *Pseudomonas aeruginosa*; res = Resistant; susc = Susceptible

The meta-analysis of unadjusted data found that use of AIAT for *P. aeruginosa* infections, as compared with IIAT significantly decreased mortality, but with heterogeneity (6 studies; summary OR 0.48, 95% CI 0.28–0.81; I^2^ = 58.6, 95% CI 0–83%) (Fig. 2). Only one study reported the outcome of in-hospital mortality [13]. The subgroup analyses identified that AIAT significantly decreased 30-day mortality, but with heterogeneity, when initiation of antibiotics was within 48 h of index blood culture (3 articles [12, 13, 16]; summary OR 0.46, 95% CI 0.32–0.67; I^2^ = 0.0, 95% CI 0–85%), in blood stream infections (3 articles [12, 15, 16]; summary OR 0.36, 95% CI 0.19–0.68; I^2^ = 43.7, 95% CI 0–81%), and among patients admitted to the hospital wards (3 articles [12, 15, 16]; summary OR 0.36, 95% CI 0.19–0.68; I^2^ = 58.6, 95% CI 0–81%). In subgroup meta-analysis by low risk of bias, AIAT was associated with lower mortality for *P. aeruginosa* infections (3 articles; unadjusted summary OR 0.46, 95% CI 0.28–0.81; I^2^ = 89.7, 95% CI 0–85%), while meta-analysis of unclear risk of bias showed AIAT was not associated with lower mortality for *P. aeruginosa* infections (2 articles; unadjusted summary OR 0.45, 95% CI 0.08–2.62; I^2^ = 89.7, 95% CI NA) compared to IIAT. Meta-regression found no relationship between age (in years) or percent of patients with ICU admissions and a decrease in mortality with AIAT.

#### Adjusted data

Two studies reported an adjusted association between IIAT and mortality in *P. aeruginosa* infections and found a significantly increased mortality with IIAT, as compared with AIAT (Adjusted hazard ratio [HR] 2.95, 95% CI 1.63–5.33 [13]; adjusted HR 5.54, 95% CI 2.15–14.56 [15]). The third study reported that receipt of IIAT significantly increased the multivariable adjusted odds of carbapenem resistant *P. aeruginosa* [14].

### Risk factors for acquiring resistant *P. aeruginosa*

Nineteen studies reported in 17 articles (two articles contributed to four studies) were included (Table 3) [12, 14–16, 18–30]. A total of 3203 total subjects (1077 cases and 2126 controls) were examined for risk factors of acquisition of MDR and resistant *P. aeruginosa*. Five studies (286 cases of 882 total patients) reported data on MDR *P. aeruginosa* [12, 18, 19, 22, 30]; 14 studies (12 articles) reported on resistant *P. aeruginosa* (791 cases of 2321 total patients)*.* Included studies were conducted in Brazil (13 studies [12, 14–16, 18, 21, 23–28, 30]), Colombia (3 studies [19, 20, 22]), and Cuba (1 study [29]). Six studies recruited case patients from ICU and the remaining studies included patients admitted to the hospital (Table 3). Risk of bias for these studies is presented in Supplemental Table 4.

**Table 3:** Study characteristics and results of risk factors for acquiring *P. aeruginosa*

Author Year	Country (Enrollment Period)	Study Design	Hospital Setting	Type of Funding	Total N	Site of Infection	Source of infection	Case / Exposure	Control / comparator	Mean Age yr	% Male
Araujo 2016 [12]	Brazil (2009–2012; 2014)	CC	Academic	Gov	236	Bacteremia 100%	Nosocomial and Community-acquired	MDR-PA	Non-MDR PA	52.7	70.3
Cortes 2009 [22]	Colombia (2001–2002)	CC	Gov	NR	96	Operation site 31%, RS 31%, BSI 50%	Nosocomial	MDR-PA	Random patients hospitalized the same day	43.5	51
DalBen 2013 [23]	Brazil (2000–2002)	PC	Tertiary	Gov; Academic	325	NR	Nosocomial	CRPA	NA	44	41
Dantas 2014 [15]	Brazil (2009–2011)	RC	Academic	Gov	120	BSI 100%	NR	Resistant,MDR-PA and XDR-PA	NA	51.5	63.3
Fortaleza 2006 (Study 1) [25]	Brazil (1992–2002)	CC	Academic	NR	324	Wound 21.3%, BSI 19.4%, UTI 16.7%	Nosocomial	IRPA or Ceftazidine-resistant PA	Patients without IRPA who were admitted to the same ward	44.3	63
Fortaleza 2006 (Study 2) [25]	Brazil (1992–2002)	CC	Academic	NR	165	UTI 27.3%, RS 25.5%, wounds 21.8%,	NR	IRPA or Ceftazidine-resistant PA	Patients without IRPA who were admitted to the same ward	42.3	66
Furtado 2009 [28]	Brazil (2003–2004)	CC	Academic	NR	245	UTI 34.9%; RS 22.2%; catheter tip 20.6%	Nosocomial	IRPA	Patients hospitalized in the same unit and matched to case patients	Median: Cases: 50, Controls: 54	61.6
Furtado 2010 [24]	Brazil (2006–2008)	CC	Academic	NR	295	RS 100%	Nosocomial	IRPA	Patients without PA receiving care in same ICU	54	59.3
Gomes 2012 [30]	Brazil (2002–2007)	PCC	Tertiary	Gov	60	NR	Nosocomial	MDR-PA	Controls	Median: Cases: 50, Controls: 40	66.7
Medell 2012 [29]	Cuba (2011)	PC	Tertiary	Gov	12	VAP 100%	Nosocomial	PA	NA	55.5	NR
Neves 2010 [18]	Brazil (2004–2005)	Ecological design; RC	Academic	NR	350	NR	NR	MDR-PA	NA	NR	NR
Ossa-Giraldo 2014 [19]	Colombia (2009–2010)	CC	Academic	University Hospital	140	NR	Nosocomial	MDR-PA	Susceptible PA	43.3	70
Pereira 2008 [27]	Brazil (2000–2002)	CC	Academic	Academic	59	UTI 60%, BSI 7%, RS 17%	Nosocomial	IRPA	ISPA	51.3	62.7
Rossi 2017 [14]	Brazil (2009–2012)	CC	Academic	University Hospital	157	Unknown: 62.42; RS: 17.19; BSI: 13.37	Nosocomial	CRPA	CSPA	66.9	31.2
Royer 2015 [21]	Brazil (2011–2012)	PC	Academic	Gov	30	VAP 100%	Nosocomial	CRPA	NA	58.97	80
Tuon 2012 [16]	Brazil (2006–2009)	CC	Tertiary	NR	77	BSI 100%	Nosocomial	CRPA	CSPA	47.4	23.7
Valderrama 2016 [20]	Colombia (2008–2014)	CC	Academic	University Hospital	168	RS 30%; GI 26%; Primary 13.7%	Nosocomial	CRPA	CSPA	Cases: 60; Controls: 64.5	53
Zavascki 2005 (Study1) [26]	Brazil (2002–2003)	CC	Tertiary	Gov	186	IRPA:RS 33.4%, UTI 26.9%, Control: NR	Nosocomial	IRPA	Random patients from same unit	54.5	56.5
Zavascki 2005 (Study2) [26]	Brazil (2002–2003)	CC	Tertiary	Gov	158	IRPA: RS 33.4%, UTI 26.9%, Control: NR	Nosocomial	IRPA	ISPA	54.7	62.7

*BSI* Bacteremia; *CC* Case-control; *CRPA* Carbapenem resistant *Pseudomonas aeruginosa*; *CSPA* Carbapenem susceptible *Pseudomonas aeruginosa*; *Gov* Government; *GI* Gastrointestinal; *IRPA* imipenem-resistant *Pseudomonas aeruginosa*; *ISPA* imipenem- susceptible *Pseudomonas aeruginosa*; *MDR* Multi-drug resistant; *N* Number; *NA* Not applicable; *NR* Not reported; *PA Pseudomonas aeruginosa*; *PC* Prospective; *PCC* Prospective case-control; *RC* Retrospective; *RS* Respiratory; *UTI* Urinary tract infection; *VAP* ventilator-associated pneumonia; *XDR* Extreme drug resistant; *Yr* year

The descriptions of study control groups were sparse across studies: susceptible *P. aeruginosa* (8 studies); non- *P. aeruginosa* (1 study); and no specific data was reported in 10 studies. No meta-analysis was performed owing to the lack of common comparator pathogen.

#### Results of studies of MDR *P. aeruginosa*

All five studies of MDR *P. aeruginosa* examined prior use of antibiotics, two studies examined ICU stay, and one study each examined a variety of risk factors: co-occurring disease, surgical procedure, hospital stay, inappropriate therapy, enteral feeding, parenteral feeding, mechanical ventilation, and female sex (Table 4). We did not perform meta-analysis owing to the small number of eligible studies for any particular comparison.

**Table 4:** Risk for Predicting Acquisition of Multi-drug Resistant *Pseudomonas aeruginosa*

Risk Category	Risk Factor	Author Year	N MDRPA	N Control	Control description	Multivariate Results (95%LCI,95%UCI); ***P***-value
**Co-occurring condition**	Diabetes Mellitus	Araujo 2016 [12]	96	140	Non-MDR PA	*OR 1.9907 (0.97,4.09); 0.0608*
**Procedure**	Surgery	Cortes 2009 [22]	24	72	Control	*OR 2.8 (0.7,11.7); 0.14*
	Mechanical ventilation					OR 12.2 (0.1,12.6); 0.014
**Stay**	Hospital	Ossa-Giraldo 2014 [19]	70	70	Susceptible PA	OR 1.03 (1.01,1.05); sig
	ICU	Cortes 2009 [22]	24	72	Control	*OR 1.2 (0.6,12.7); 0.91*
	ICU	Dantas 2014^a^ [15]	57	65	Resistant PA	OR 3.28 (NR); 0.02
**Therapy**	IIAT	Araujo 2016 [12]	96	140	Non-MDR PA	OR 3.0169 (1.72,5.31); 0.0001
**Feeding**	Parenteral feeding	Gomes 2012 [30]	15	45	Non-MDR PA	OR 10.7 (1.5,91.9); 0.018
	Enteral feeding	Gomes 2012 [30]	15	45	Non-MDR PA	OR 14.9 (3.3,94.1); 0.003
**Prior antibiotic use**	Aminoglycoside	Neves 2010 [18]	81	269	Non-MDR PA	Correlation coefficient 0.31 (NR); 0.14
	Aminoglycoside	Ossa-Giraldo 2014 [19]	70	70	Susceptible PA	OR 3.09 (1.26,7.58); NR
	Carbapenem	Neves 2010 [18]	81	269	Non-MDR PA	Correlation coefficient 0.67 (NR); 0.01
	Carbapenem	Araujo 2016 [12]	96	140	Non-MDR PA	OR 0.8928 (0.51,1.55); 0.6873
	Quinolone	Neves 2010 [18]	81	269	Non-MDR PA	Correlation coefficient − 0.13 (NR); 0.57
	Quinolone	Gomes 2012 [30]	15	45	Non-MDR PA	OR 8.9 (1.6,66.4); 0.013
	3rd-generation Cephalosporin	Neves 2010 [18]	81	269	Non-MDR PA	Correlation coefficient 0.16 (NR); 0.31
	>2antimicrobials for > 48 h in prior 30 d	Ossa-Giraldo 2014 [19]	70	70	Susceptible PA	OR 4.4 (1.1,17.65); NR
	Any	Cortes 2009 [22]	24	72	Control	OR 2.7 (1.2125); 0.19
**Sex**	Female	Ossa-Giraldo 2014 [19]	70	70	Susceptible PA	OR 2.31 (1.02,5.2); sig
**Source of infection**	Respiratory tract source of bacteremia	Dantas 2014^a^ [15]	57	65	Resistant PA	5.83 (NR); 0.02

^a^ Both MDRPA and XDRPA

*ICU* Intensive care unit; *LCI* Lower confidence interval; *MDRPA* Multi-drug Resistant; *N* Number; *NR* Not reported; *OR* Odds ratio; *PA Pseudomonas aeruginosa*; *UCI* Upper confidence interval

Two studies each reported that previous use of aminoglycoside, carbapenem, and fluorquinolones was a significant predictor for acquisition of MDR *P. aeruginosa.* In one study, all variables were significant (Table 4), except for the presence of type II diabetes, undergoing surgical procedure, and ICU stay.

#### Results of studies of Resistant *P. aeruginosa*

Of 14 studies in 12 articles, the significant predictors of acquisition of resistant *P. aeruginosa* were prior use of antibiotics (9 studies), stay in the ICU (3 studies), and comorbidity score (3 studies). Significant predictors were reported in at least two studies for each of the following: co-occurring diseases, hospital stay, hemodialysis, length of stay, male sex, and parenteral nutrition. Other significant predictors reported in at least one study included: mechanical ventilation, inappropriate therapy, colonization pressure, admission diagnosis, surgical procedure, use of corticosteroids, respiratory infection, and transfer from another hospital.

Nine studies (in 11 data points) reported prior use of different classes of antibiotics as a significant predictor for acquisition of resistant *P. aeruginosa.* The most frequently assessed antibiotics included carbapenem and aminoglycosides (Supplemental Table 5). All of the examined predictors were reported as significant predictors for acquisition of resistant *P. aeruginosa* except for the following in three studies: Acute Physiology And Chronic Health Evaluation II (APACHE II) and hospital stay (1 study [20]), heart failure as a co-occurring disease (1 study [21]), and number of antibiotics used (1 study [24]).

### GRADE rating of overall strength of evidence

The overall GRADE rating showed that there was low strength of evidence across outcomes that were primary and/or most frequently reported in eligible studies (Supplemental Table 6), These included mortality for the comparisons of AIAT with IIAT (primary outcome), and the most frequently reported outcomes of previous use of antibiotics, comorbidity score, and hospital stay as a risk factor for the acquisition of MDR or resistant *P. aeruginosa*, and hospital stay and APACHE II as a risk factor for the acquisition of resistant *P. aeruginosa.* All the aforementioned outcomes were graded to be of low strength of evidence owing to risk of bias (unclear or high) and imprecise estimates.

## Discussion

This systematic literature review and meta-analysis of studies from the LATAM region identified several important findings among hospitalized adult patients with *P. aeruginosa.* First, AIAT compared with IIAT was associated with a halving of 30-day mortality (unadjusted summary OR = 0.48). This significance persisted in subgroup analyses, when appropriate antibiotics were initiated within 48 h, in blood stream infections, and among patients admitted to the hospital wards. Although there were no studies to draw conclusions for < 24-h or 24--to-48-h time points, this review demonstrates that a 48-h time point is a possible threshold beyond which a significantly higher mortality risk is observed with further delays in AIAT.

Second, the choice of an initial antibiotic therapy against a possible *P. aeruginosa* infection has been a challenge owing, in part, to different resistance trends, mechanisms of resistance and evolution of resistance. For example, resistance to the carbapenem class, an important class of initial therapy in severe nosocomial infections, is now reported to be more than 70% in some hospitals in the LATAM region [32]. Indeed, a number of individual studies exist in the public domain examining risk factors associated with resistant *P. aeruginosa* infections in LATAM. Notably, prior use of antibiotics (11 studies), stay in the ICU (3 studies), and comorbidity score (3 studies) were significant predictors of acquisition of resistant *P. aeruginosa*.

The mechanisms of resistance and variation in resistance based on the type of antibiotics can present some complexities in targeting antibiotic therapy – involving either acquisition of genes against beta-lactams and aminoglycosides, or mutation of chromosomal genes in the case of resistance against fluoroquinolones [33]. These challenges may result in delays in the initiation and administration of appropriate antibiotics that can lead to adverse patient outcomes. An understanding of local epidemiology specific to the LATAM region is essential in early diagnosis and administration of AIAT.

The development of resistance can also evolve over time. For example, *P. aeruginosa* has evolved virulence characteristics that may make it a difficult target for antibiotic therapy. Resistant strains of *P. aeruginosa* are the most frequent source of HAIs and are often associated with overuse or inappropriate use of antimicrobials. Our review highlights that there was a consistent association between prior use of antimicrobials and acquisition of resistant *P. aeruginosa*. Our findings concur with many other studies that showed exposure to previous antimicrobial therapy prior to the development of current infection can lead an increased risk of resistance to different classes of antibiotics [34]. A thorough understanding of risk factors associated with acquisition of resistant *P. aeruginosa* can aid in the monitoring of resistant pathogens in order to prevent emergence of MDR/XDR/pan-drug resistant pathogens. More importantly, knowledge about these risk factors can help tailor presumptively better antimicrobials to be used.

This review and meta-analysis have several strengths. To the best of our knowledge, there was no previously published systematic review and meta-analysis from the LATAM region comparing AIAT with IIAT and systematically reviewed studies from the LATAM region evaluating multivariate risk factors predicting acquisition of *P. aeruginosa*. This review included studies published in English, Spanish and Portuguese languages from the LATAM region. Additionally, this review comprehensively searched for studies indexed in regional databases. The overall results from this review are consistent with other systematic reviews published literature from other regions that compare the role of AIAT with IIAT in mortality [8, 35, 36]. Nonetheless, in light of a few limitations, the results of the systematic review and meta-analysis should be interpreted with caution. There was a general lack of studies reporting adjusted analyses for mortality outcomes. Therefore, our meta-analysis is restricted to unadjusted results comparing AIAT with IIAT that did not account for potential confounders. Overall, risk of bias among individual studies was high and there was inconsistencies across studies. The majority of studies were retrospective observational studies. This translated into a low overall strength of evidence that was confirmed by a formal evaluation of the GRADE to assess the overall strength of evidence. The impact of AIAT versus IIAT on treatment response, length of stay, and costs in the LATAM region is unknown because there were no studies evaluating these outcomes. In addition, studies either did not clearly define AIAT and IIAT, or lacked consistent definitions on timeliness and susceptibility to antimicrobial therapy.

The following gaps in the literature merit consideration for future research. There is a need to examine outcome measures including costs, length of stay, clinical and microbiologic success in studies published from the LATAM region in future studies. Data on the risk factors predicting acquisition of *P. aeruginosa* were generally inconsistent or not particularly highlighting any particular factors. The limitations of this review as well as any future reviews will reflect, to a large extent, the limitations of the data in primary studies. Therefore, future, well-conducted, well-analyzed (preferably prospective) observational studies are warranted in this area.

## Conclusion

This systematic literature review and meta-analysis suggests significantly decreased 30-day mortality with AIAT, as compared with IIAT among patients hospitalized with *P. aeruginosa* infections in the LATAM region. This review also highlights that use of prior antibiotics, co-morbid diseases or severity scores, and prior ICU/hospital stay as consistent and significant risk predictors of acquisition of resistant *P. aeruginosa* among hospitalized patients from the LATAM region.

This review synthesizes important evidence from the LATAM region, which to the best of our knowledge, was not systematically assessed before. This evidence that may make a significant impact on clinical and health policy decision-making. The policy implementation of our findings in the LATAM region would benefit by engaging infectious disease specialists, hospitalists, epidemiologists, and other relevant stakeholders. AIAT against a possible infection has been a challenge owing, in part, to different resistance trends in several regions of the world. In addition to implementation of infection control programs in hospitals, a thorough understanding of local epidemiology and better laboratory facilities are essential in targeting appropriate antibiotic. Given the high resistance rates in the LATAM region and the worse outcomes associated with *P. aeruginosa* infections, there is a continued need for the use of appropriate antimicrobial agents with activity against *P. aeruginosa*. The consistent role of prior use of antibiotics or a stay in ICU in the acquisition of resistant *P. aeruginosa* reinforces the importance of antimicrobial stewardship programs, especially in the ICU setting among critically ill patients.

## Supplementary information

**Additional file 1 Table S1.** Search Strategy comparing AIAT vs. IIAT. **Table S2.** Search Strategy for acquisition of resistant *P. aeruginosa*. **Table S3.** Risk of Bias Assessment for AIAT vs. IIAT Studies. **Table S4.** Risk of Bias Assessment for Risk Factors Studies. **Table S5.** Risk Factors Predicting Acquisition of Resistant *P. aeruginosa*. **Table S6.** GRADE Overall Strength of Evidence for Relevant Outcomes.

## Data Availability

Data supporting the conclusions of this article is available in the Supplementary Material, figures, and tables. Detailed data extraction forms are available upon request from the corresponding author.

## Abbrevations

AHRQ: Agency for Healthcare Research and Quality
AIAT: Appropriate initial antibiotic therapy
APACHE II: Acute Physiology And Chronic Health Evaluation II
BIREME: Biblioteca Regional de Medicina
CI: Confidence Interval
ES: Effect size
ESBL: Extended-spectrum beta-lactamases
GRADE: Grading of Recommendations Assessment, Development and Evaluation
HAIs: Healthcare associated infections
HR: Hazard Ratio
ICU: Intensive Care Unit
IIAT: Inappropriate initial antibiotic therapy
LATAM: Latin American countries
LILAC: Literatura Latino Americana em Ciências da Saúde
MDR: Multi-drug Resistant
NA: Not applicable
OR: Odds Ratio
PACTS: Program to Assess Ceftolozane/Tazobactam Susceptibility
PRISMA: Preferred Reporting Items for Systematic Reviews and Meta-Analyses
XDR: Extremely drug-resistant
