# Supplementary material for: Pseudomonas infections among hospitalized adults in Latin America: a systematic review and meta-analysis
Source: BMC Infect Dis. 2020 Mar 27;20:250. doi: 10.1186/s12879-020-04973-0 (PMC7099820; doi:10.1186/s12879-020-04973-0)
Supplement: Supplementary file 1 — Additional file 1 Table S1. Search Strategy comparing AIAT vs. IIAT. Table S2. Search Strategy for acquisition of resistant P. aeruginosa. Table S3. Risk of Bias Assessment for AIAT vs. IIAT Studies. Table S4. Risk of Bias Assessment for Risk Factors Studies. Table S5. Risk Factors Predicting Acquisition of Resistant P. aeruginosa. Table S6. GRADE Overall Strength of Evidence for Relevant Outcomes. [file 12879_2020_4973_MOESM1_ESM.doc]

**SUPPLEMENTAL TABLES**

**Supplemental Table 1. Search Strategy comparing AIAT vs. IIAT**

| **#** | **Searches** |
| --- | --- |
| 1 | exp urinary tract infection$/ and complicat$.af. |
| 2 | (urinary and tract and infection$ and complicat$).tw. |
| 3 | (exp pyelonephritis/ or pyelonephritis.tw.) and complicat$.af. |
| 4 | exp intraabdominal infections/ and complicat$.af. |
| 5 | (intraabdominal and infection$ and complicat$).tw. |
| 6 | (intra-abdominal and infection$ and complicat$).mp. or (intra-abdominal and abscess).tw. [mp=title, abstract, original title, name of substance word, subject heading word, keyword heading word, protocol supplementary concept word, rare disease supplementary concept word, unique identifier] |
| 7 | ((ruptur$ or perforat$ or complicat$) and (appendicitis or cholecystitis or diverticulitis or peritonitis or intestine or typhilits)).tw. |
| 8 | (exp blood circulation/ and exp infections/) or (bloodstream and infection$).tw. or (bacteremia or bacteraemia).tw. |
| 9 | (exp pneumonia/ or pneumonia.tw. or pneumoniae.tw.) and (ventilator-associated or (ventilator and associated)).tw. |
| 10 | (respiratory and infection$ and complicat$).af. |
| 11 | (VAP or BSI or IAI or UTI or HAP or HCAP).tw. |
| 12 | or/1-11 |
| 13 | exp Pseudomonas/ or Pseudomonas.tw |
| 14 | or/13-16 |
| 15 | 12 and 17 |
| 16 | *Inappropriate Prescribing/ and (empiric or initial).af. |
| 17 | appropriate.af. or appropriate.tw. |
| 18 | inappropriate.af. or inappropriate.tw. |
| 19 | adequate.af. or adequate.tw. or adequacy.tw. or effective.tw. |
| 20 | or/16-19 |
| 21 | (Anti-bacterial or anti-infective or antibiotic$ or antimicrobial).af. and (dt or tu or th).fs. |
| 22 | Anti-Bacterial Agents/ad or Anti-Bacterial Agents/tu or Cross Infection/dt or Anti-Infective Agents/tu or Antibiotic$.af. or Drug Therapy, Combination/tu |
| 23 | 21 or 22 |
| 27 | 20 and 26 |
| 28 | 18 and 27 |
| 29 | (nosocomial or hospital-acquired or healthcare-acquired or hospital-associated or healthcare-associated or hospital or hospitals or hospitalized or (intensive and care) or (critical and care)).af. |
| 30 | 28 and 29 |
| 31 | (review or letter or case reports or editorial or comment).pt. |
| 32 | 30 not 31 |
| 33 | (anguilla or aruba or antigua) and barbuda) or argentina or bahamas or Guyana or netherland antilles or nicaragua or panama or paraguay or paraguai or (saint kitts and nevis) or (saint vincent and the grenadines) or suriname or trinidad) and tobago) or (turks and caicos) or West indies or Suriname or uruguay or venezuela or west indies or brazil or argentina or chile or Colombia or Bolivia or peru or Ecuador or Paraguay or Guiana or curacao or caribbean or saint marten or saint lucia or Dominican republic or haiti or dominica or jamaica or cayman or Monstserrat or Anguilla or belize or mexico or nicaragua or honduras or costa rica or panama or guatemala or El salvador or cuba or Guadeloupe or Martinique or Puerto rico or saint barthelemy or virgin islands).af. |
| 34 | 32 and 33 |

**Supplemental Table 2. Search Strategy for acquisition of resistant P. aeruginosa**

| **#** | **Searches** |
| --- | --- |
| 1 | exp Pseudomonas infection/di, dr, ep, et |
| 2 | exp antibiotic resistance/ |
| 3 | exp multidrug resistance/ |
| 4 | 2 or 3 |
| 5 | 1 and 4 |
| 6 | (MDR or XDR or PDR).tw. |
| 7 | 1 and 6 |
| 8 | acquisition.tw. |
| 9 | 5 and 8 |
| 10 | (MDR-PA or XDRPA or PDRPA or MR-PA or PDR-PA).tw. |
| 11 | 5 or 7 or 9 or 10 |
| 12 | cross infection/ or hospital-acquired.mp. |
| 13 | hospital/ or hospital infection/ or nosocomial.mp. |
| 14 | catheter infection/ or healthcare associated infection/ or healthcare-associated.mp. |
| 15 | Emergency service, hospital/ |
| 16 | exp intensive care/ |
| 17 | ICU.tw. |
| 18 | ((critical or intensive) and care).tw. |
| 19 | or/12-18 |
| 20 | 11 and 19 |
| 21 | predictive value of tests/ |
| 22 | risk assessment/ |
| 23 | multivariate analysis/ |
| 24 | risk factors/ |
| 25 | logistic models/ |
| 26 | predict:.tw. |
| 27 | (risk and scor:).tw. |
| 28 | scor:.tw. |
| 29 | validat:.tw. |
| 30 | or/21-29 |
| 31 | 20 and 30 |
| 32 | (anguilla or aruba or antigua) and barbuda) or argentina or bahamas or Guyana or netherland antilles or nicaragua or panama or paraguay or paraguai or (saint kitts and nevis) or (saint vincent and the grenadines) or suriname or trinidad) and tobago) or (turks and caicos) or West indies or Suriname or uruguay or venezuela or west indies or brazil or argentina or chile or Colombia or Bolivia or peru or Ecuador or Paraguay or Guiana or curacao or caribbean or saint marten or saint lucia or Dominican republic or haiti or dominica or jamaica or cayman or Monstserrat or Anguilla or belize or mexico or nicaragua or honduras or costa rica or panama or guatemala or El salvador or cuba or Guadeloupe or Martinique or Puerto rico or saint barthelemy or virgin islands).af. |
| 33 | 31 and 32 |

**Supplemental Table 3.** Risk of Bias Assessment for AIAT vs. IIAT Studies

| **Author Year** | **Selection bias: Is the source population (cases, controls, cohorts) appropriate and representative of the population of interest?** | **Performance bias: Is the sample size adequate and is there sufficient power to detect a meaningful difference in the outcome of interest?** | **Detection bias: Did the study use appropriate statistical analysis methods relative to the outcome of interest?** | **Detection bias: Missing data handling was addressed clearly** | **Information bias: Is the methodology of the outcome measurement explicitly stated and is it appropriate?** | **Was the outcome measure objectively?** |
| --- | --- | --- | --- | --- | --- | --- |
| Araujo 2016 | Low | Low | unclear | unclear | Low | Low |
| Dantas 2014 | unclear | High | unclear | unclear | Low | Low |
| Gonzales 2014 | unclear | Low | Low | Low | Low | Low |
| Pinheiro 2008 | Low | unclear | High | unclear | Low | Low |
| Rossi Gonclaves 2017 | Unclear | Low | Low | Unclear | Low | Low |
| Tuon 2012 | Low | High | Low | unclear | Low | Low |

**Supplemental Table 4. Risk of Bias Assessment for Risk Factors Studies**

| **Author Year** | **Selection bias: Is the source population (cases, controls, cohorts) appropriate and representative of the population of interest?** | **Performance bias: Is the sample size adequate and is there sufficient power to detect a meaningful difference in the outcome of interest?** | **Detection bias: Did the study use appropriate statistical analysis methods relative to the outcome of interest?** | **Detection bias: Missing data handling was addressed clearly** | **Information bias: Is the methodology of the outcome measurement explicitly stated and is it appropriate?** | **Was the outcome measure objectively?** |
| --- | --- | --- | --- | --- | --- | --- |
| Araujo 2016 | Low | Low | Unclear | Unclear | Low | Low |
| Cortes 2009 | Low | Unclear | Unclear | Unclear | Low | Low |
| DalBen 2013 | Low | Low | Unclear | Low | Low | Low |
| Dantas 2014 | Unclear | High | Unclear | Unclear | Low | Low |
| Fortaleza 2006 (Study1) | Low | High | Low | Unclear | Low | Low |
| Fortaleza 2006 (Study2) | Low | High | Low | Unclear | Low | Low |
| Furtado 2009 | Low | High | Low | Unclear | Low | Low |
| Furtado 2010 | Unclear | High | Unclear | Unclear | Low | Low |
| Gomes 2012 | Low | High | High | Unclear | Low | Low |
| Medell 2012 | Unclear | High | High | Unclear | Low | Low |
| Neves 2010 | Low | Unclear | High | Unclear | Low | Low |
| Ossa-Giraldo 2014 | Unclear | Low | High | High | Low | Low |
| Pereira 2008 | Low | High | Unclear | Unclear | Low | Low |
| Rossi Gonclaves 2017 | Unclear | Low | Low | Unclear | Low | Low |
| Royer 2015 | Unclear | High | High | Unclear | Unclear | Low |
| Tuon 2012 | Low | High | Low | Unclear | Low | Low |
| Valderrama 2016 | Low | Low | Low | High | Low | Low |
| Zavascki 2005 (Study1) | Low | Low | High | Unclear | Low | Low |
| Zavascki 2005 (Study2) | Low | High | High | Unclear | Low | Low |

**Supplemental Table 5. Risk Factors Predicting Acquisition of Resistant P. aeruginosa**

| **Risk Categories** | **Multivariable Risk Factors** | **Author Year** | **N PA** | **N Control** | **Risk for acquisition of** | **Control** | **Multivariate Results (95%LCI, 95%UCI); P-value** |
| --- | --- | --- | --- | --- | --- | --- | --- |
| **Colonization pressure** | Colonization pressure in the week before the outcomes | DalBen 2013 | 67 | 258 | MRAC and CRPA | non MRAC/CRPA | OR 1.02 (1.01, 1.04); 0.008 |
| **Co-morbidity score** | APACHE II | Valderrama 2016 | 42 | 126 | CRPA | CSPA | OR 1.02 (0.95, 1.09); 0.58 |
|  | APACHE II score | DalBen 2013 | 67 | 258 | MRAC and CRPA | non MRAC/CRPA | OR 1.11 (1.06, 1.16); <0.001 |
|  | APACHE II score | Furtado 2010 | 58 | 237 | IRPA | ISPA | OR 1.11 (1.01, 1.22); 0.003 |
| **Co-occuring condition** | Heart failure | Royer 2015 | 15 | 30 | P. aeruginosa | A. baumannii | OR 0.6279 (0.14, 2.88); 0.549 |
|  | Hemodialysis | Royer 2015 | 15 | 30 | P. aeruginosa | A. baumannii | OR 0.1424 (0.03, 0.7); 0.017 |
|  | Hemodialysis | Furtado 2010 | 58 | 237 | IRPA | ISPA | OR 6.85 (1.33, 35.2); 0.02 |
|  | Renal failure | Zavascki(b) 2005 | 93 | 65 | IRPA | ISPA | OR 5 (1.28, 19.53); 0.02 |
| **Feeding** | Parential nutrition | Valderrama 2016 | 42 | 126 | CRPA | CSPA | OR 8.28 (2.56, 26.79); <0.001 |
|  | Probes enteral/gastric nutrition | Goncalves 2017 | 69 | 88 | CRPA | Susceptible PA | OR 3.8347 (1.43, 10.26); 0.0074 |
| **Hospital procedure** | Surgery prior to admission | DalBen 2013 | 67 | 258 | MRAC and CRPA | non MRAC/CRPA | OR 0.29 (0.13, 0.65); 0.003 |
|  | Mechanical ventilation | Zavascki(a) 2005 | 93 | 93 | IRPA | Non-PA | OR 3.22 (1.52, 6.83); 0.002 |
|  | urinary catheter >15 days (collinear variable) | Pereira 2008 | 30 | 29 | IRPA | ISPA | OR 5.95 (1.59, 22.33); <0.01 |
| **IIAT** | Inappropriate therapy | Goncalves 2017 | 69 | 88 | CRPA | Susceptible PA | OR 8.5534 (3.28, 22.32); <0.0001 |
| **Other** | Clinical diagnosis at admission | Royer 2015 | 21 | 30 | P. aeruginosa | A. baumannii | OR 0.2671 (0.06, 1.11); 0.069 |
| **Other meds** | Use of corticosteriod | Furtado 2010 | 58 | 237 | IRPA | ISPA | OR 13.18 (3.8, 45.64); <0.001 |
| **Prior AB use** | Aztreonam | Medell 2012 | 12 |  | P. aeruginosa | Non-PA | NR (NR, NR); 0.003 |
|  | Ciprofloxacin | Valderrama 2016 | 42 | 126 | CRPA | CSPA | OR 81.99 (1.14, 58.84); 0.043 |
|  | Piperacillin-tazobactam | Furtado 2010 | 58 | 237 | IRPA | ISPA | OR 14.31 (1.02, 200.16); 0.04 |
|  | 3rd-gen cephalosporin | Furtado 2010 | 58 | 237 | IRPA | ISPA | OR 7.45 (1.8, 30.86); 0.006 |
|  | Amikacin use | Fortaleza (a) 2006 | 108 | 216 | IRPA | Control | adj OR 3.22 (1.4, 7.41); 0.005 |
|  | Amikacin | Fortaleza (b) 2006 | 55 | 110 | Ceftazidine-resistant PA | Control | OR 3.69 (1.32, 10.35); 0.01 |
|  | No. of antibiotics used | Furtado 2009 | 63 | 182 | IRPA | Control | OR 1.38 (0.97, 1.97); 0.07 |
|  | Meropenem | Valderrama 2016 | 42 | 126 | CRPA | CSPA | OR 1.15 (1.03, 1.28); 0.01 |
|  | Carbapenem (including ertapenem) | Tuon 2012 | 29 | 48 | CRPA | CSPA | OR NR (NR, NR); 0.014 |
|  | Carbapenem use irrespective of vancomycin | Zavascki(a) 2005 | 93 | 93 | IRPA | Non-PA | OR 5.82 (2.41, 4.07); <0.001 |
|  | Carbapenem use without vancomycin | Zavascki(a) 2005 | 93 | 93 | IRPA | Non-PA | OR 3.57 (1.38, 9.19); 0.008 |
|  | Carbapenem use with vancomycin | Zavascki(a) 2005 | 93 | 93 | IRPA | Non-PA | OR 43.71 (4.46, 428.53); <0.001 |
|  | Carbapenem use irrespective of vancomycin | Zavascki(b) 2005 | 93 | 65 | IRPA | ISPA | OR 12.82 (3.99, 41.23); <0.001 |
| **Prior admission** | At least one admission in last year | Zavascki(a) 2005 | 93 | 93 | IRPA | Non-PA | OR 2.59 (1.2, 5.56); 0.015 |
| **Procedure** | Mechanical ventilation | Goncalves 2017 | 69 | 88 | CRPA | Susceptible PA | OR 3.0674 (1.22, 7.73); 0.0175 |
| **Sex** | Male gender | DalBen 2013 | 67 | 258 | MRAC and CRPA | non MRAC/CRPA | OR 2.24 (1.24, 4.05); 0.008 |
|  | Male gender | Furtado 2010 | 58 | 237 | IRPA | ISPA | OR 8.01 (1.66, 38.51); 0.009 |
| **Source of infection** | Primary bacteremiawith unknown focus | Goncalves 2017 | 69 | 88 | CRPA | Susceptible PA | OR 0.2363 (0.08, 0.73); 0.0127 |
| **Stay** | Hospital stay | Valderrama 2016 | 42 | 126 | CRPA | CSPA | OR 1.02 (0.98, 1.05); 0.33 |
|  | Length of hospital stay | Furtado 2010 | 58 | 237 | IRPA | ISPA | OR 1.19 (1.12, 1.26); <0.001 |
|  | Hospital stay >15 days | Pereira 2008 | 30 | 29 | IRPA | ISPA | OR 6.7 (1.86, 24.14); <0.01 |
| **Stay-ICU** | Hospitalization in the ICU unit | Valderrama 2016 | 42 | 126 | CRPA | CSPA | OR 0.95 (0.92, 1); 0.031 |
|  | Length in ICU care before VAP (>7) | Royer 2015 | 20 | 30 | P. aeruginosa | A. baumannii | OR 0.2913 (0.08, 1.11); 0.071 |
|  | Previous ICU stay | Furtado 2009 | 63 | 182 | IRPA | Control | OR 3.54 (1.29, 9.73); 0.03 |
| **Transfer** | Transfer from another hospital | Fortaleza (b) 2006 | 55 | 110 | Ceftazidine-resistant PA | Control | OR 18.61 (2.04, 174.28); 0.01 |

**Supplementary Table 6.** GRADE Overall Strength of Evidence for Relevant Outcomes

|  |  |  |  |  |  |  |  | **Summary of Findings** | | |
| --- | --- | --- | --- | --- | --- | --- | --- | --- | --- | --- |
| **Outcome** | **# of Studies & Study Design** | **Total N Patients** | **Risk of Bias** | **Consistency across studies** | **Precision** | **Directness of the Evidence** | **Other Considerations** | **Quality of Evidence** | **Effect size** | **Description of Effect size** |
| **Unadjusted Mortality AIAT vs. IIAT in *P. aeruginosa*** | 6 studies in 5 articles (All retrospective) | 728 | 3 Low;  2 unclear | Serious inconsistency | Imprecise | Direct | None* | **Low** | Summary OR 0.51  95% CI 0.32–0.82 | AIAT will likely decrease mortality in P. aeruginosa infection compared to IIAT |
| **Adjusted Mortality AIAT vs. IIAT in *P. aeruginosa*** | 2studies  (All RO) | 284 | 1 Low;  1 unclear | No inconsistency | Imprecise | Direct | None | **Low** | Adjusted HR 2.95, 95% CI 1.63–5.33  Adjusted HR 5.54, 95% CI 2.15–14.56 | AIAT will likely decrease mortality in P. aeruginosa infection compared to IIAT |
| **Prior use of antibiotics as a risk factor for MDR *P. aeruginosa*** | 5 studies  (1 PC; 4 RO) | 882 | 1 Low; 3 unclear; 1 High | Serious inconsistency | Imprecise | Direct | None | **Low** | OR ranged between 0.89 and 8.9 | Prior use of antibiotics was likely a risk factor for MDR *P. aeruginosa* compared to control |
| **Prior use of antibiotics as a risk factor for resistant *P. aeruginosa*** | 9 studies  (1 PC; 8 RO) | 1630 | 6 Low, 2 High, 1 unclear | No inconsistency | Imprecise | Direct | Incomplete reporting in 2 of 9 studies | **Low** | OR ranged between 1.15 and 81.99 | Prior use of antibiotics was likely a risk factor for resistant *P. aeruginosa* compared to control |
| **Comorbidity score as a risk factor for resistant *P. aeruginosa*** | 3 studies  (1 PC; 2 RO) | 788 | 2 Low; 1 unclear | No inconsistency | Imprecise | Direct | None | **Low** | OR ranged between 1.02 and 1.11 | APACHE II score was likely a risk factor for resistant *P. aeruginosa* compared to control |
| **Hospital stay as a risk factor for resistant *P. aeruginosa*** | 3 studies | 463 | 2 Low; 1 unclear | No inconsistency | Imprecise | Direct | None | **Low** | OR ranged between 1.02 and 6.7 | Stay in the hospital was likely a risk factor for resistant *P. aeruginosa* compared to control |

* No publication bias was assessed as less than 10 studies were available for this outcome

CI = confidence interval; HR = hazard ratio; OR = odds ratio; PC = prospective cohort; RO = retrospective observational
